# Effects of the combinations of amlodipine/valsartan versus losartan/hydrochlorothiazide on left ventricular hypertrophy as determined with magnetic resonance imaging in patients with hypertension

**DOI:** 10.3109/21556660.2011.639418

**Published:** 2011-12-16

**Authors:** Oliver Bruder, Christoph J. Jensen, Michael Bell, Reinhard Rummel, Guenter Boehm, Sven Klebs, Christian Sieder, Jochen Senges

**Affiliations:** 1 Department of Cardiology and Angiology, Elisabeth Hospital, Essen, Germany; 2 Office-based cardiologist, Berlin, Germany; 3 Office-based internist, Ludwigshafen, Germany; 4 Novartis Pharma GmbH, Clinical and Regulatory Affairs, Nürnberg, Germany; 5 Institute for Myocardial Infarction Research, Ludwigshafen, Germany

**Keywords:** Arterial hypertension, left ventricular hypertrophy, end-organ damage, magnetic resonance imaging, treatment, combination therapy, RAAS, Asc. aorta, ascending aorta, A/V, amlodipine plus valsartan, CCB, calcium channel blockers, BP, diastolic blood pressure, IVS, interventricular septum thickness, LA, left atrium, L/H, losartan/hydrochlorothiazide, LVEDV, left ventricular enddiastolic volume, LVEF, left ventricular ejection fraction, LVESV, left ventricular endsystolic volume, LVH, left ventricular hypertrophy, LVM, left ventricular mass, LVMI, left ventricular mass index, MRI, magnetic resonance imaging, norm BSA, adjusted for body surface area, SBP, systolic blood pressure

## Abstract

**Background:**

Left ventricular hypertrophy (LVH), a marker of cardiac end-organ damage, is frequently found in patients with arterial hypertension and is associated with cardiovascular and cerebrovascular morbidity and mortality. Therefore, LVH regression is an important treatment goal. For amlodipine plus valsartan (A/V) no specific study on LVH has been reported to date.

**Methods:**

Prospective, open-label, randomized parallel-group study. Patients with essential hypertension and LVH were randomized to 52-week treatment with A/V 10/160 mg (*n* = 43) or the active comparator losartan/HCT 100/25 mg (L/H, *n* = 47). Add-on medication was allowed in case of inadequate blood pressure control. LV parameters were measured by cardiovascular magnetic resonance imaging (MRI), and adjudicated in a blinded manner. Study identifiers were NCT00446563 and EudraCT 2006-001977-17.

**Results:**

In addition to the study treatment, 35% of patients in the A/V group and 49% in the L/H group received additional antihypertensive medication. Compared to baseline, both treatments reduced measures of LVH significantly after 52 weeks (e.g. LV mass index in the A/V group from 64.7 g/m^2^ by −3.5 g/m^2^, in the L/H group from 69.1 g/m^2^ by −4.4 g/m^2^, *p* < 0.01 for both). LV ejection fraction and LV volumes were not significantly changed by any regimen. A/V and L/H treatments were well tolerated.

**Conclusions:**

Both regimen were effective in reducing LV mass compared to baseline and were well tolerated.

## Introduction

The main cardiac response to primary hypertension is left ventricular hypertrophy (LVH), which is found in at least 30% of patients.^1,2^ As a marker of cardiac end-organ damage, LVH has been recognized to robustly predict cardiovascular and cerebrovascular complications in the general population and patients with hypertension more strongly than other risk factor except for advancing age.^3,4^ These findings have provoked great interest in how regression of LVH can be achieved and whether different antihypertensives differ in their ability to reduce myocardial hypertrophy in addition to lowering blood pressure.^5^

Several large meta-analyses with several thousand patients examined the ability of antihypertensive drugs on the reversal of LVH, and found very consistent effects.^6–8^ Schmieder *et al.* did an analysis of 50 randomized double-blind studies published until 1996. They reported that overall for any active treatment left ventricular mass index (LVMI) was the more reduced the greater the decrease in systolic blood pressure, (*r* = 0.27; *p* < 0.05), the longer the duration of therapy (*r* = 0.36; *p* < 0.001), and the higher the pretreatment value of left ventricular mass index (*r* = 0.53; *p* < 0.001).^7^ According to a more recent meta-analysis of the same group - including 80 randomized double-blind studies - LVMI decreased by 13% with angiotensin II receptor antagonists, by 11% with calcium antagonists, by 10% with ACE inhibitors, by 8% with diuretics, and by 6% with beta-blockers.^8^

One of the presumed mechanisms is that RAAS blockade exerted by renin inhibitors, ACE inhibitors, and angiotensin II receptor blockers inhibits the effects of angiotensin II (stimulation of myocyte cell growth) and aldosterone (increase of collagen content and stimulation development of myocardial fibrosis).^5,9,10^

In recent years increasing interest has been laid on the effect of combination therapy on LVH, as the majority of patients with moderate or severe hypertension require two or more drugs in order to achieve target blood pressure levels.^11–14^ Calcium channel blockers (CCB) plus angiotensin II receptor blockers (ARB) have complimentary mechanisms of action, are among recommended combinations,^15,16^ and are increasingly used in clinical practice.^17^ The dihydropyridine amlodipine, at doses of 5–10 mg once daily, is one of the most frequently used CCBs, and is indicated also for chronic stable or vasospastic angina pectoris.^18,19^ The ARB valsartan, approved at doses of 80–320 mg once daily, is an effective and well-tolerated once-daily antihypertensive agent, with a tolerability profile similar to placebo.^20,21^ The drug is also indicated for patients with heart failure or post myocardial infarction.^21^ In the VALUE trial, valsartan was shown to reduce the risk of developing new-onset diabetes in hypertensive patients at high risk of cardiac events compared with CCB treatment.^22^ Further, in diabetic patients with microalbuminuria, valsartan has been shown to have benefits beyond those attributable to blood pressure lowering alone.^21^ To date, no study has been published on the effect of combined treatment with amlodipine/valsartan (A/V) on measures of LVH. Losartan was chosen as comparator, as a losartan-based regimen showed favorable effects on LVH in the LIFE study.^23^

The present study was performed to investigate (1) whether treatment with A/V 10/160 mg in free combination reduces LVH in patients with mild-to-moderate hypertension, and (2) whether in the head-to-head comparison of A/V versus losartan plus HCT 100/25 mg (L/H) one treatment is superior over the other.

## Patients and methods

### Study design

This was a randomized, multicenter, open-label parallel group study in 12 recruiting centers in Germany. It was approved by the ethics committee in Rhineland-Palatinate and the responsible health authority (BfArM, Federal Institute for Drugs and Medical Devices). The trial was conducted according to Good Clinical Practice.

### Patients

Male and female adult patients aged 18–80 years with essential hypertension were eligible for inclusion, if they had the following diastolic blood pressure values:

- if not treated with an antihypertensive drug: DBP 95–110 mmHg (at Visit 1),
- if treated with 1 drug: DBP 90– 110 mmHg,
- if treated with 2 or 3 drugs: DBP 90–105 mmHg. Patients on 3 antihypertensive drugs could only be included, if not all drugs were given at the highest approved dose level.

Further, patients had to present with LVH: LV wall thickness 12–16 mm determined by the highest value either by the posterior wall thickness or by the interventricular septal wall thickness confirmed by echocardiography at Visit 2 prior to randomization. In case of confirmed LVH, the MRI was performed and patients were randomized.

Key exclusion criteria included: secondary forms of hypertension; severe refractory hypertension (SBP > 180 mm Hg or DBP of >110 mm Hg at any visit); hypertrophic cardiomyopathies due to etiologies other than hypertension; history of symptomatic heart failure (NYHA classes II-IV) or a LVEF < 50% confirmed by echocardiography prior to randomization; history of stroke, transient ischemic cerebral attack, hypertensive encephalopathy, coronary artery bypass surgery, percutaneous transluminal angioplasty or myocardial infarction any time prior to visit 1; concurrent life threatening arrhythmia or symptomatic arrhythmia; 2nd or 3rd degree heart block, sick sinus syndrome or sinuatrial block; hepatic disease or cholestasis (AST or ALT values exceeding 3×upper limit of normal); type 1 diabetes mellitus or poorly controlled treated type 2 diabetes mellitus; known or suspected contraindications for MRI (e.g. pacemakers; defibrillators; inability to lie supine).Women of child-bearing potential had to use a highly effective method of birth control.

### Procedures

**Figure 1.**
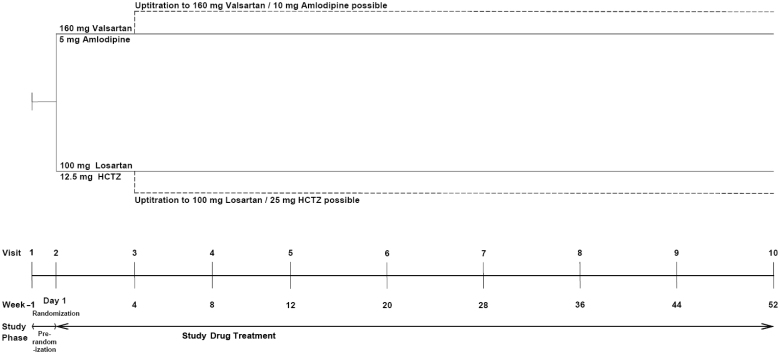
Study design.

The study consisted of 3 phases, as shown in Figure 1. During Screening, patients were assessed for eligibility with regards to inclusion and exclusion criteria. Patients were screened at sites which had no option to perform an MRI as well as at sites which had such equipment (local MRI/echocardiography centers). Patients were allowed to continue their current medication until randomization. If tapering off was necessary, it had to be ensured that these medications were tapered off until Visit 3, the latest.

Period 2 (Baseline Titration) starting with Visit 2: At visit 2 patients where seen in selected centers that were eligible to perform echocardiography and MRIs. When the qualifying level of LVH was given, the baseline MRI was conducted. An independent person for the randomization process at the center who was unaware of any clinical results of baseline measurements provided the unique randomization number to the patient which allocated the patient to one of the treatment arms with either A/V 5/160 mg or L/H 100/12.5 mg for 4 weeks.

Period 3 (Maintenance) started with Visit 3, at which patients achieving their target blood pressure of mean sitting SBP/DBP < 140/90 mmHg were kept on their original treatment, or were to be titrated to the maximum allowed daily dose of either A/V 10/160 mg or L/H 100/25 mg, respectively. If the target BP could not be reached with the latter regimen, at the discretion of the investigator additional antihypertensive medications (vasodilators or selective alpha_1_ receptor blockers) were allowed at Visit 4 or later. Total duration of active treatment was 52 weeks.

### Study drugs

Drugs were administered in free combination: amlodipine mesylate (Amlodipine Sandoz 5 mg or 10 mg (manufacturer: Sandoz Pharmaceuticals, Ismaning, Germany) and since 2008, amlodipine besylate (Amlobesilat-Sandoz® 5 mg or 10 mg, manufacturer: Sandoz Pharmaceuticals, Ismaning, Germany), valsartan (Diovan® 80 mg or 160 mg film coated tables, manufacturer: Novartis Pharma GmbH, Germany), HCT (HCT Sandoz 12.5 mg or 25 mg, manufacturer: Sandoz Pharmaceuticals, Ismaning, Germany); losartan (Lorzaar® Protect 50 mg or 100 mg, manufacturer MSD Chibropharm, Haar, Germany). Study drugs had to be taken once daily in the morning.

### Efficacy parameters

This was a randomized open-label trial with merchandized drugs. Patients and study centers were not blinded to the treatment. Therefore, the raw data of the primary and secondary objectives was recorded blinded to the treatment and interpreted by the core reading center which was blinded to the treatment of the individual patients (M. Bell and C.J. Jensen). Furthermore, to avoid any bias caused by local investigators when recruiting patients, a central randomization procedure was used. The primary efficacy parameter of this trial was change from baseline of the LVMI normalized to body surface area (BSA) by MRI. Other efficacy assessments included the MRI measurements left ventricular mass (LVM), interventricular septum (IVS) thickness, left ventricular posterior wall thickness, left ventricular ejection fraction (LVEF), left ventricular enddiastolic volume (LVEDV), left ventricular endsystolic volume (LVESV), ascending aortic diameter, and left atrial area. Further, changes in pulse rate, changes in mean sitting SBP und DBP, and blood pressure responder rates were calculated.

### Safety and tolerability assessment

The safety information included Adverse Events (AE), results of physical examinations, data on weight, and laboratory evaluations. Vital signs were assessed as part of the efficacy evaluations.

An AE was defined as the appearance or worsening of any undesirable sign, symptom, or medical condition occurring after obtaining informed consent even if the event was not considered to be related to the study drug. Medical conditions/diseases present before obtaining informed consent were only considered as an AE if they worsened after the study start. Abnormal laboratory values or test results constituted an AE only if they induced clinical signs or symptoms, required study drug discontinuation or required therapy. AEs were recorded by the severity grade (mild, moderate, severe), their relationship to the study drug(s) (suspected/not suspected), their duration (start and end dates or if continuing at final examination) and whether it constituted a serious adverse event (SAE). A SAE was defined as an event which was fatal or life-threatening, resulted in persistent or significant disability/incapacity, constituted a congenital anomaly/birth defect, required inpatient hospitalization or prolongation of existing hospitalization or was medically significant.

A complete physical examination was performed at Visit 1. In addition, a standard 12-lead ECG and a urine pregnancy test in women were performed.

Body weight (with the patient in street clothes and without shoes) was measured at Visit 1 and study end. Blood pressure and pulse were measured at all visits. Laboratory tests in fasted state were performed at all visits (hematology/blood chemistry at Visit 1 and last visits, chemistry only at all other visits), and analyzed at a central laboratory.

### Blood pressure measurement

Sitting blood pressure was recorded using standard fully automatic upper-arm blood oscillometric pressure monitor with the appropriate size cuff, as provided by the sponsor. Measurements were repeated 3 times, and print-out of the measurements kept in the study files. The pulse rate was recorded during the BP measurements.

### Sample size calculation

The sample size was originally calculated to support the superiority claim and assumed a difference of 6 g/m^2^ between treatments with a common SD of 10. Under these assumptions, 60 patients per treatment arm would have been required to achieve 90% power at the 2-sided 5% significance level. To compensate for drop-outs and other protocol violations, a total number of 150 patients was planned to be randomized into this trial.

### Statistical analysis

All randomized patients who took at least one dose of study medication were included in the safety analyses as well as in the efficacy analyses. An additional per-protocol analysis included all patients who did not have major deviations from the protocol procedures that might have an impact on the study outcome.

The trial was originally designed to demonstrate the non-inferiority of the experimental treatment (combination A/V) compared with the reference treatment (L/H), and then, in a second hierarchical step, the superiority of A/V vs. L/H. Non-inferiority was defined as a difference less than 3 g/m^2^ in the change in left ventricular mass index.

However, during the conduct of the study it became apparent that despite various efforts it was not possible to recruit the patients for this study in a reasonable time-frame. It was therefore decided to stop recruitment of new patients, to allow all patients already included to complete the study. Due to the under-recruitment of the trial compared to the originally planned sample size, it was obvious that it would be underpowered, so the trial objectives were downgraded to just exploratively comparing both treatment regiments. Treatments were compared using an analysis of covariance (ANCOVA) model with the factors treatment and prior antihypertensive therapy and covariate baseline LVMI was used. The unadjusted as well as the adjusted (least square) means were calculated together with an estimate of the treatment contrast, a p-value and the 95% confidence interval for the treatment contrast. To explore the non-inferiority-, an additional, 1-sided p-value was calculated for the shifted null hypothesis that the true difference is equal to delta.

## Results

### Patient characteristics and disposition

**Figure 2.**
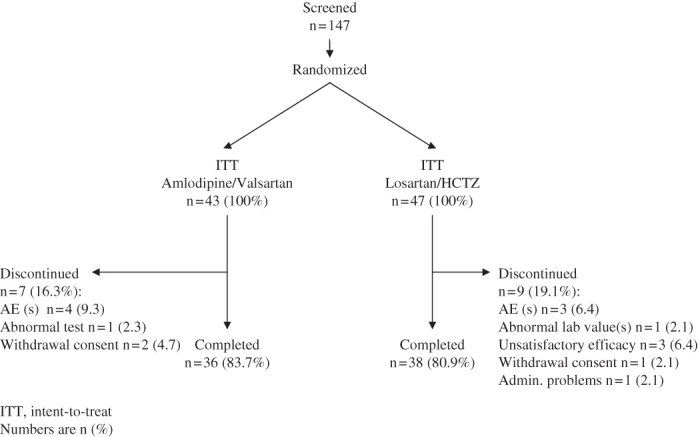
Patient flow.

The disposition of patients over the study duration is presented in Figure 2. In total, 147 patients were screened of which 90 patients (less than originally planned) were approved to undergo randomization and subsequent treatment. Seven patients discontinued treatment with A/V and nine with L/H. Thus, the study was completed by 74 patients. The safety population and ITT population were identical (90 patients), and the PP population comprised 71 patients due to one or more protocol violations in 19 patients (16 premature discontinuation, 4 forbidden concomitant medication, 2 low compliance, 1 LVH criteria missed at baseline).

Baseline characteristics of patients are summarized in Table 1. All patients were Caucasian, and there was a preponderance of males (72.2%). Mean age of patients was 57.7 ± 11.5 years, and 31.1% were aged 65 years or above. Mean SBP/DBP was 161.7/96.5 mmHg, and values were slightly higher in the L/H group compared to the A/V group. Patient characteristics in the PP set were similar to the safety/ITT set (data not shown).

**Table 1.** Baseline demographics.

Characteristics	Randomized Patients		
	A/V (*n* = 43)	L/H (*n* = 47)	Total (*N* = 90)
Age, years range	58.2 ± 12.2	57.2 ± 10.9	57.7 ± 11.5
	30–83	33–78	30–83
Gender, male,%	31 (72.1)	34 (72.3)	65 (72.2)
Height, cm	173.8 ± 9.3	172.7 ± 9.2	173.2 ± 9.2
Weight, kg	91.3 ± 19.4	90.0 ± 14.7	90.6 ± 17.0
Body Surface Area, m^2^	2.1 ± 0.3	2.0 ± 0.2	2.0 ± 0.2
Caucasian	43 (100)	47 (100)	90 (100)
Diabetes mellitus	3 (7.0)	8 (17.0)	10 (11.1)
SBP, mmHg	158.5 ± 16.8	164.7 ± 18.8	161.7 ± 18.0
DBP, mmHg	95.5 ± 9.4	97.4 ± 11.0	96.5 ± 10.2

Values are n (%), or mean value ± standard deviation. SBP, systolic blood pressure; DBP, diastolic blood pressure.

The explorative analysis of the efficacy results is presented in Table 2.

**Table 2.** Efficacy measures.

Parameter	Value at baseline		Value at 52 weeks**		Absolute change 52 weeks vs. baseline			Relative change (%) at 52 weeks vs. baseline		
	AV	LH	AV	LH	AV	LH	Diff AV/LH	AV	LH	Diff AV/LH
LVM, g	133.4 ± 32.3	141.2 ± 36.1	126.3 ± 29.3	132.1 ± 35.6	−7.1 ± 16.5*	−9.1 ± 18.9*	0.6 [−6.5; 7.7]	−4.4 ± 12.1*	−5.8 ± 13.1*	0.7 [−4.4; 5.9]
LVMI/norm BSA, g/m^2^	64.7 ± 12.4	69.1 ± 15.2	61.2 ± 10.8	64.7 ± 15.0	−3.5 ± 7.4*	−4.4 ± 9.3*	−0.1 [−3.4; 3.2]	−4.4 ± 12.1*	−5.8 ± 13.1*	0.3 [−4.9; 5.4]
IVS, mm	14.1 ± 2.2	14.6 ± 1.7	12.9 ± 1.8	13.9 ± 1.6	−1.1 ± 1.5*	−0.6 ± 1.3*	−0.7 [−1.3;−0.1]*	−7.1 ± 9.5*	−3.9 ± 8.5*	−4.1 [−8.1; 0.0]
Posterior wall, mm	10.8 ± 1.7	11.2 ± 1.7	10.3 ± 1.7	10.9 ± 1.7	−0.4 ± 1.1*	−0.3 ± 1.2	−0.2 [−0.7; 0.3]	−3.1 ± 9.8*	−1.8 ± 9.7	−2.0 [−6.6; 2.6]
LVEF, %	67.7 ± 7.6	68.3 ± 7.1	67.2 ± 6.5	67.4 ± 7.0	−0.8 ± 6.7	−0.4 ± 5.9	−0.3 [−2.9; 2.3]	−0.4 ± 10.7	−0.1 ± 9.3	−0.1 [−4.0; 3.8]
LVEDV, ml	127.5 ± 35.8	130.1 ± 40.7	126.3 ± 35.5	116.9 ± 37.6	0.1 ± 19.9	−6.4 ± 22.9	7.1 [−2.7; 16.9]	1.7 ± 17.3	−3.0 ± 20.1	5.2 [−3.4; 13.7]
LVEDV norm, ml/m^2^	61.9 ± 14.9	63.8 ± 18.6	61.9 ± 16.0	57.5 ± 16.0	0.0 ± 9.6	−3.0 ± 11.2	3.4 [−1.4; 8.1]	1.7 ± 17.3	−3.0 ± 20.1	5.3 [−3.2; 13.7]
LVESV, ml	43.1 ± 18.0	41.9 ± 17.6	43.0 ± 16.9	39.0 ± 17.9	0.4 ± 10.3	−1.5 ± 10.3	2.3 [−2.3; 7.0]	4.2 ± 26.2	−0.5 ± 29.3	5.6 [−7.4; 18.5]
LVESV norm, ml/m^2^	20.7 ± 7.8	20.5 ± 8.2	20.9 ± 7.9	19.1 ± 7.9	0.2 ± 5.1	−0.8 ± 5.1	1.2 [−1.1; 3.5]	4.2 ± 26.2	−0.5 ± 29.3	5.6 [−7.3; 18.4]
LA, cm^2^	20.1 ± 4.5	21.3 ± 5.1	20.1 ± 4.8	19.9 ± 4.3	−0.6 ± 3.2	−1.0 ± 4.5	0.4 [−1.4; 2.1]	−2.0 ± 17.1	−3.1 ± 21.5	0.7 [−8.1; 9.5]
Asc. aorta, mm	35.8 ± 3.4	38.4 ± 4.8	36.1 ± 4.4	38.3 ± 5.6	0.1 ± 1.9	−0.8 ± 1.6	1.1 [−0.5; 2.6]	0.1 ± 5.3	−2.0 ± 4.4	2.7 [−1.7; 7.0]

Asc. aorta, ascending aorta; norm BSA, adjusted for body surface area; diff, difference; LA, left atrium; IVS, interventricular septum thickness; LVEDV, left ventricular enddiastolic volume; LVEF, left ventricular ejection fraction; LVESV, left ventricular endsystolic volume; LVM, left ventricular mass; LVMI, left ventricular mass index. *p < 0.05, **Absolute changes are given in the left column units by parameter.

### Primary endpoint: left ventricular mass index

Mean (unadjusted) LVMI significantly decreased in the A/V group from 64.7 g/m^2^ to 61.2 g/m^2^ at study end (absolute change −3.5 g/m^2^, relative change −4.4%), and in the L/H group significantly from 69.1 to 64.7 (absolute change −4.4 g/m^2^, relative change −5.8%). The absolute difference between groups was −0.1 g/m^2^ (95% CI −3.4; 3.2), the relative change was 0.3% (−4.9; 5.4). The effect in the PP were similar (absolute change 0.1 gm^2^ [−3.7; 3.8]; relative change 0.0% [−5.9; 6.0]). Differences between groups were not significant in either analysis population.

### Secondary endpoints

Mean IVS was significantly reduced by both treatments (A/V −1.1 mm; L/H −0.6 mm), and the resulting difference between groups in favour of A/V in the ITT population was significant for the absolute change (−0.7 mm, 95% CI −1.3; −0.1), and showed a trend for significance for the relative change (−4.1%; 95% CI −8.1; 0.0).

Posterior wall thickness was reduced significantly by A/V but not by L/H vs. baseline but the difference between groups did not reach statistical significance.

At the end of the study, within the two groups there were no significant changes noted for LVEF, LVEDV as well as LVESV (unadjusted or normalised by BSA), LA area or diameter of the ascending aorta. The difference between the two treatment groups was also not statistically significant.

### Blood pressure

In addition to the study treatment, 35% of patients in the A/V group and 49% in the L/H group received additional (“rescue”) antihypertensive medication.

Mean SBP/DBP decreased in the A/V group from 158.5/95.5 to 142.6/85.6 (change from baseline −15.7/−10.3 mmHg), in the L/H group from 164.7/97.4 mmHg to 153.5/91.9 mmHg (change from baseline −10.7/−5.4 mmHg). The difference between both treatments at week 52 was statistically significant (*p* = 0.0051 for systolic BP, *p* = 0.0121 for diastolic BP) but the change over the whole study duration was not (*p* = 0.26 for systolic BP, *p* = 0.07 for diastolic BP). Pulse increased in the A/V group from baseline to final visit by 1.4 beats per minute, in the L/H group it remained the same.

The proportion of patients with blood pressure <140/90 mmHg at study end without additional antihypertensive medication was 64% in the A/V group and 25% in the L/H group (*p* = 0.0061). When additional antihypertensive medication was considered, BP normalisation at study end was achieved by 54% in the A/V group and 15% in the L/H group (*p* = 0.0002).

### Tolerability and Safety

Overall, a total of 173 AEs was reported in 62 patients (30 patients in the A/V group, 32 in the L/H group). 17 SAEs occurred in 8 patients (2 patients in the A/V group, 6 in the L/H group). In the V/A group, one patient presented a CUP syndrome, hydrocele testis left and cholecystitis as SAEs, and one patient presented coronary heart disease progression, coronary 3 vessel disease with vascular obliteration, and myocardial infarction. In the L/H group one patient presented hypertriglyceridemia and a thalamus infarction as SAEs, one patient the worsening of coxarthrosis, one patient presented anal bleeding, one patient presented diarrhea, abdominal pain and a colon carcinoma, one patient bradycardia, tachycardia, hypokalemia (the only SAE with suspected relationship to study drug), and neuritis vestibularis, and one patient presented with erysipelas as SAE. No death occurred.

AEs with suspected drug relation were relatively infrequent and balanced across groups. Frequencies within each body system are summarized in Table 3. In total, nasopharyngitis was reported as the AE with the highest frequency (10 patients in total, thereof 3 in the V/A group and 7 in the L/H group), followed by back pain (7 patients in total, thereof 5 in the V/A group and 2 in the L/H group), and diarrhoea (6 patients in total, thereof 1 in the V/A group and 5 in the L/H group).

**Table 3.** Number of patients with Adverse Events (AEs), by system organ class.

	A/V (*n* = 43)	L/H (*n* = 47)
	*n* (%)	*n* (%)
**Adverse Events, total number**	87	86
Patients with AE(s)	30 (69.8)	32 (68.1)
with suspected drug relation	8 (18.6)	7 (14.9)
leading to dose adjustment or temp. interruption	1 (2.3)	0
leading to permanent discontinuation	4 (9.3)	4 (8.5)
requiring concomitant medication/non-drug therapy	25 (58.1)	24 (51.1)
**Serious Adverse Events (SAEs), total number**	5 (5.7)	12 (14.0)
Patients with SAEs	2 (4.7)	6 (12.8)
Deaths	0	0
SAEs with suspected drug relation	0	1 (2.1)
SAEs leading to permanent discontinuation	1 (2.3)	2 (4.3)
**AEs by system organ class**	**n (%)**	**n (%)**
Cardiac disorders	3 (3.4)	3 (3.5)
Congenital, familial and genetic disorders	1 (1.1)	0
Ear and labyrinth disorders	0	4 (4.7)
Eye disorders	0	0
Gastrointestinal disorders	6 (6.9)	17 (19.8)
General disorders and administration site conditions	8 (9.2)	4 (4.7)
Hepatobiliary disorders	1 (1.1)	0
Immune system disorders	0	1 (1.2)
Infections and infestations	22 (25.3)	18 (20.9)
Injury, poisoning and procedural complications	6 (6.9)	2 (2.3)
Investigations	0	2 (2.3)
Metabolism and nutrition disorders	3 (3.4)	6 (7.0)
Musculoskeletal and connective tissue disorders	14 (16.1)	10 (11.6)
Neoplasms benign, malignant and unspecified (including cysts and polyps)	1 (1.1)	1 (1.2)
Nervous system disorders	7 (8.0)	5 (5.8)
Psychiatric disorders	0	2 (2.3)
Reproductive system and breast disorders	0	1 (1.2)
Renal and urinary disorders	0	0
Respiratory, thoracic and medistinal disorders	6 (6.9)	2 (2.3)
Skin and subcutaneous tissue disorders	5 (5.7)	4 (4.7)
Surgical and medical procedures	0	0
Vascular disorders	4 (4.6)	4 (4.7)
*n*, number of patients,		

## Discussion

The present study evaluated the effects of two different open label treatments on left ventricular hypertrophy reduction during 1-year treatment. According to the exploratory analysis of the present study, combination therapy with either A/V or L/H led to reduction of LVH parameters by MRI compared to baseline values. The reduction in the A/V group was similar than in the L/H group. However, due to the limited sample size, noninferiority could not be demonstrated.

Despite randomisation, blood pressure values in the study were notably higher in the L/H group at baseline. The study protocol stipulated that blood pressure values should be normalized in both groups, by adding additional antihypertensive drugs as needed. Indeed add-on antihypertensive medication was more frequent in the L/H group (49% compared to 35% in the A/V group); however, it did not lead to the extent of blood pressure control as planned. In view of the high proportion of patients needing add-on drugs, the current results have been found for A/V- and L/H-*based* therapy rather than true double combination therapy. However, as many patients receive three or even more drugs to control hypertension,^24^ this approach closely emulates clinical practice. Even if the blood pressure levels at baseline as well as the change in blood pressure from baseline to study end were not statistically different between the two treatment groups, the reduction in systolic blood pressure was numerically more pronounced with A/V, and statistically significantly more patients reached the target blood pressure in the A/V group. On the other hand, the numerically higher LV mass index at baseline may have been in favour of L/H to achieve a more pronounced LVH regression.^7^ Thus, differences in baseline MRI, baseline blood pressure levels and the amount of antihypertensive add-on treatments may have influenced the outcomes of MRI measurements.

For all 4 antihypertensive drugs used in this study, previous studies - mostly using echocardiography - have reported favourable effects on LVH. For *amlodipine* at doses of 5–10 mg, Fak *et al.* reported that in 30 mild to moderate essential hypertensive patients with diastolic dysfunction LVMI decreased significantly from 160 ± 30 to 137 ± 26 g/m^2^ at 3 months and remained stable at 6 months.^25^ Islim *et al.* noted in a 20-week, open-label, noncontrolled study in 12 per protocol patients a significant regression in LVMI (from 169.0 ± 30.7 g/m^2^ to 140.6 ± 19.6 g/m^2^).^26^ Further studies support these findings, e.g. a comparison with irbesartan (LVMI decreased by 23.2% in the irbesartan-treated patients and by 11.4% in the amlodipine-treated patients).^27^

Beneficial effects of *valsartan* were reported by Thürmann *et al.* in 58 patients,^28^ by Mutlu *et al.* in 30 patients^29^, by Gottdiener *et al.*,^30^ and by Suzuki *et al.* (in type 2 diabetics)^31^. Picca *et al.* in 2004 reported a head-to-head comparison of valsartan 160 mg with losartan 100 mg in a small cohort of patients (*n* = 30) with untreated hypertension and concentric LVH. After 6-month therapy, the reduction in left ventricular mass index assessed by echocardiography was significantly greater in the valsartan group than in the losartan group.^32^

With regard to *HCT*, Okin *et al.* reported that the agent was used in >70% of patients in the LIFE study. HCT was associated with greater regression of LVH by ECG and this effect was greater in patients on losartan- than atenolol-based therapy, independent of baseline severity of LVH, hypertension and changes in BP.^33^ In a double-blind comparison, 14-month HCT was significantly less effective than 18-month enalapril in LVH.^34^

Finally, for *losartan*, the Losartan Intervention for Endpoint Reduction in Hypertension (LIFE) study was the largest prospective, randomized double-blind LVH trial to date. It demonstrated a superior reduction of cardiovascular mortality and morbidity as well as a significant regression of LVM by angiotensin II blockade with losartan in combination with HCT compared to the combination of atenolol with HCT.^35^ In an echo substudy (*n* = 960), the losartan-based regimen significantly reduced LVMI after 36 weeks compared with baseline (−6.6 g/m^2^, 95% CI −10.2 to −2.9 g/m^2^, *p* < 0.001), whereas the atenolol-based regimen had no significant effect (−3.7 g/m^2^, −7.8 to 0.3 g/m^2^, *p* = n.s.).^36^ The estimated treatment difference between the losartan and atenolol regimens (mean change from baseline at week 36) in LVMI was −2.5 g/m^2^ (95% CI −7.4 to 2.4 g/m^2^ in favour of losartan), indicating that losartan was significantly non-inferior (*p* < 0.001, non-inferiority limit 8 g/m^2^) and numerically superior to atenolol in reducing LVMI.

Given the manifold differences in LVH studies indirect comparisons of results can be misleading. Potential differences with respect to patient characteristics, treatment strategies (e.g. monotherapy vs. various combination approaches, titration ranges of drugs), measurements of LVH (electrocardiography, echocardiography, MRI), follow-up durations must be taken into account. Overall, treatment effects in our study were in the same magnitude as in other current MRI based studies, for example the ALLAY study of aliskiren vs. losartan vs. aliskiren/losartan (reductions of LVMI between 4.9 to 5.8 g/m^2^).^37^

We used cardiovascular MRI for the quantification of LVM, as it is considered the gold standard for this purpose. Compared to M-mode or 2-dimensional echocardiography, MRI has much greater accuracy, greater reproducibility and less variability.^38^ It generates a spatially defined 3-dimensional dataset at multiple contiguous levels throughout the heart, hence, the measurement of left ventricular mass is not based on geometric assumptions about the left ventricle, and leads to excellent agreement between MRI-obtained and true LVMs.^9^ However, a number of methodological issues remain controversial, including whether to include or exclude papillary muscles and LV trabeculae in the LVM estimation^39^ and how to best index LVM measurements to body size^37^. Compared to echocardiography, CMR studies need much smaller sample sizes: Bottini *et al.* demonstrated that to detect a decrease of 10 g LVM (power 80% at *p* = 0.05) required 550 patients by echocardiography, but only 17 patients on MRI.^40^

Further methodological considerations have to be taken into account. As the difficulties in recruitment and the resulting recruitment stop led to a patient number lower than planned in the sample size calculation, the analyses should be regarded as exploratory. The study was randomized and active controlled, which minimizes bias. A placebo control would in principle have been preferable to verify the drug-induced effects, but is from an ethical perspective not acceptable over a long period. The 1-year study duration was probably not long enough to show the full effect of treatment on LVH. In the LIFE study beyond the substantial decrease in LVM during the first year, especially in losartan-treated patients, there were smaller further decreases in LV wall thicknesses, relative wall thickness, and LVMI during years 2 and 3 in both treatment arms.^23^ These results suggest that the benefit of antihypertensive treatment on LV remodelling cannot be fully appreciated unless treatment trials last at least 3 years.^23^

In our study, A/V and L/H were investigated as free combinations since the A/V fixed dose combination was not yet available at study start. In clinical practice, single pill combinations, specifically calcium channel blocker/ARB combinations, have been found to be associated with improved compliance and persistence vs. free combinations of the individual components.^41^ Eventually, improved compliance and persistence are associated with a higher probability to achieve blood pressure targets,^42^ a lower risk for hospitalizations due to cardiovascular events,^43^ and a reduced utilization of medical resources.^44^ These findings give raise to the assumption effects on LVH may also be greater if single-pill combinations rather than free combinations are applied.

In terms of tolerability, only a minority of patients on A/V or L/H combinations had AEs with a suspected relationship to study drug according to the investigator. Also after the addition of further antihypertensive medication, i.e., triple combination therapy, tolerability was good. The general safety profile of the drugs did not differ from that in the clinical studies as reported in the respective prescribing information, or from the substantial every-day clinical experience obtained in recent years.^45,46^

## Conclusion

In this first exploratory study evaluating effect of the A/V combination in approved doses on LVH in patients with essential hypertension, this regimen was effective and well tolerated as was the comparator L/H. The data do not provide any evidence of a relevant difference between the two treatment regiments with regard to the primary endpoint.

## Transparency section

### Declaration of financial/other relationships

J.S. reports receiving lecture fees from Novartis. S.K. and C.S. are employees of Novartis Pharma GmbH Germany.

Registration. The study was registered in Clinicaltrials.gov (identifier: NCT00446563) and the EU Clinical Trial Register (EudraCT number: 2006-001977-17).
